# Impaired Latent Inhibition in GDNF-Deficient Mice Exposed to Chronic Stress

**DOI:** 10.3389/fnbeh.2017.00177

**Published:** 2017-10-10

**Authors:** Mona Buhusi, Colten K. Brown, Catalin V. Buhusi

**Affiliations:** Interdisciplinary Program in Neuroscience, Department of Psychology, Utah State University, Logan, UT, United States

**Keywords:** c-Fos, chronic stress, glial-derived neurotrophic factor, latent inhibition, nucleus accumbens, schizophrenia

## Abstract

Increased reactivity to stress is maladaptive and linked to abnormal behaviors and psychopathology. Chronic unpredictable stress (CUS) alters catecholaminergic neurotransmission and remodels neuronal circuits involved in learning, attention and decision making. Glial-derived neurotrophic factor (GDNF) is essential for the physiology and survival of dopaminergic neurons in substantia nigra and of noradrenergic neurons in the locus coeruleus. Up-regulation of GDNF expression during stress is linked to resilience; on the other hand, the inability to up-regulate GDNF in response to stress, as a result of either genetic or epigenetic modifications, induces behavioral alterations. For example, GDNF-deficient mice exposed to chronic stress exhibit alterations of executive function, such as increased temporal discounting. Here we investigated the effects of CUS on latent inhibition (LI), a measure of selective attention and learning, in GDNF-heterozygous (HET) mice and their wild-type (WT) littermate controls. No differences in LI were found between GDNF HET and WT mice under baseline experimental conditions. However, following CUS, GDNF-deficient mice failed to express LI. Moreover, stressed GDNF-HET mice, but not their WT controls, showed decreased neuronal activation (number of c-Fos positive neurons) in the nucleus accumbens shell and increased activation in the nucleus accumbens core, both key regions in the expression of LI. Our results add LI to the list of behaviors affected by chronic stress and support a role for GDNF deficits in stress-induced pathological behaviors relevant to schizophrenia and other psychiatric disorders.

## Introduction

Stress initiates integrated organismal responses, ranging from biochemical, endocrine and immune processes to behavioral alterations, in order to adapt and ensure the survival of the individual. Acute stress usually induces adaptive time-limited responses, i.e., rapid detection of threat through reallocation of resources to a network promoting vigilance, at the cost of the executive network, adequate fight-or-flight responses and restoration of homeostasis when threats are no longer present. On the other hand, persistent changes resulting from long-term chronic stress can have deleterious implications for the health and survival of the organism (De Kloet et al., 2005; Pardon and Marsden, 2008; Herman, 2013).

Cognitive dysfunction is a hallmark of chronic stress in both humans (Lupien et al., 2007; Marin et al., 2011) and experimental animals (Holmes and Wellman, 2009; Moreira et al., 2016). In rodents, chronic stress impairs performance in spatial learning and memory tasks, novel object recognition (for review see Moreira et al., 2016), behavioral flexibility (Hurtubise and Howland, 2017; Jett et al., 2017) and decision making (Dias-Ferreira et al., 2009; Buhusi et al., 2016).

The biological underpinnings of stress-induced behavioral modifications are related to the effects of stress hormones (CRF, glucocorticoids) and changes in neurotransmission (Linthorst and Reul, 2008; Joels and Baram, 2009; Herman, 2013). Chronic stress is associated with impaired glutamatergic neurotransmission (Jett et al., 2017) and altered inhibitory GABA responses in the prefrontal cortex (McKlveen et al., 2016), as well as changes in *dopamine* (DA; Ahmad et al., 2010; Belujon and Grace, 2015) and norepinephrine release (Arnsten, 2011; Jett and Morilak, 2013).

Two recent studies (Uchida et al., 2011; Bian et al., 2012) identified a major role for the *Glial-derived neurotrophic factor* (GDNF) in response to *chronic unpredictable stress* (CUS): Increased GDNF expression in the *nucleus accumbens* (Acb) and the hippocampus promotes resilience and recovery from CUS. Instead, individuals who cannot up-regulate GDNF during stress exhibit anxiety, anhedonia and avoidance of social interactions, possibly due to the negative consequences of chronic stress on the DA circuits. GDNF, a neurotrophic factor, is particularly important for the physiology of catecholaminergic neurons. GDNF and its receptors are required for neuronal development (Strömberg et al., 1993; Burke, 2006), the expression of Tyrosine Hydroxylase (the enzyme required for catecholamine synthesis; Beck et al., 1996) and the DA Transporter (required for high affinity DA uptake; Lin et al., 1993), the survival of DA neurons in the substantia nigra (Granholm et al., 2000; Boger et al., 2006; Pascual et al., 2008; Zaman et al., 2008), and the survival of noradrenergic neurons in the locus coeruleus (Zaman et al., 2003; Quintero et al., 2004; Pascual et al., 2011). Acb-derived GDNF is a retrograde enhancer of DA tone in the mesocorticolimbic system (Wang et al., 2010). Several lines of genetically engineered mice have been developed to explore the role of GDNF and its receptors in DA neuron development and survival (Pichel et al., 1996; Kramer et al., 2007; Pascual et al., 2008).

A recent study (Knapman et al., 2010) revealed that mice highly reactive to stress exhibit reversal learning and *latent inhibition* (LI) deficits. LI is defined as the loss of future associability by a stimulus that has been repeatedly presented without consequence (Lubow and Moore, 1959). LI results in slower learning of a new *conditioned stimulus* (CS)—*unconditioned stimulus* (US) association, when the *pre-exposed* (PE) stimulus is afterwards presented with consequences.

Given that LI is a process highly dependent on DA (Young et al., 2005; Weiner and Arad, 2009; Arad and Weiner, 2010), which in turn is regulated by GDNF levels, here we tested the hypothesis that stressed *GDNF-deficient* (*heterozygous*, HET) mice would be less able to increase levels of GDNF (due to having a single functional allele, Griffin et al., 2006) than their *wild-type* (WT) littermates, with negative consequences on DA function and deficits in LI. We also comparatively evaluated neuronal activation (c-Fos+ cell counts) in brain regions known to be important for LI expression—Acb and ventral hippocampus (vHipp)—in GDNF HET mice and their WT littermates.

## Materials and Methods

### Subjects

The subjects were 52 3–4 month-old male GDNF-deficient (HET, *n* = 26) mice and their WT (*n* = 26) littermate controls from a GDNF colony (Granholm et al., 1997) maintained on C57BL/6J background for at least 10 generations. Genotypes were confirmed by PCR amplification from tail biopsy samples. The mice were further divided into *Stress* (S, *n* = 26) and *No-Stress* (NS, *n* = 26) groups. Mice were housed in a temperature-controlled room under a 12-h light-dark cycle. Mice were maintained at 85% of their *ad libitum* weights by restricting access to food (Teklad Diet 8604, Envigo, Denver, CO, USA). All experimental procedures were conducted in accordance with the standards for the ethical treatment and approved by Utah State University IACUC Committee.

### Chronic Unpredictable Stress (CUS)

Stress mice received 21 days of CUS as in Dias-Ferreira et al. (2009), using the following daily randomly-chosen stressors: 30 min restraint, 10 min forced swim, or 10 min exposure to an aggressive Balb/c male mouse. We have chosen this 3-week CUS protocol since stressed C57Bl/6J mice seem to be resilient to this CUS (e.g., Buhusi et al., 2016), and the aim was to comparatively evaluate GDNF-deficient mice relative to their WT littermates. Please note that when exposed to a longer (8-week), more complex CUS protocol (Monteiro et al., 2015), C57Bl/6J mice do show changes in anxiety, depressive-like and exploratory behaviors.

### Apparatus

The apparatus consisted in eight standard mouse operant chambers housed inside sound-attenuating cubicles (Med Associates, St. Albans, VT, USA) equipped with a house light, a fan, two nosepokes on the front wall and one nosepoke on the back wall, a programmable audio generator, a shocker/scrambler module, a lever, and a standard mouse 20-mg pellet feeder. The *pre-exposed* (PE) and *non-pre-exposed* (NPE) conditioned stimuli were a 80-dB tone and a 10-Hz click. The US was a 1-s 0.5 mA footshock.

### Latent Inhibition (LI)

LI was assessed using an “on baseline” *conditioned emotional response* (CER) procedure consisting of baseline, pre-exposure, conditioning, rebaseline and test phases (i.e., allowing the mouse to eat during the all stages of the LI paradigm; Buhusi et al., 2017). Mice were assigned either to a PE tone/NPE click or PE click/NPE tone in a counterbalanced manner. Mice were shaped to nosepoke for food pellets on an FR1 schedule throughout the LI task, which consisted of four daily sessions as follows: During the 60-min pre-exposure session mice received 40 30-s presentations of the PE stimulus separated by a 60-s *inter-stimulus* interval (ISI). During the 30-min conditioning session, the PE and NPE stimuli were presented for 30 s three times, separated by a 240-s ISI. The last presentation of the PE and NPE stimuli was paired with a 1-s, 0.5-mA footshock. On the next day mice were given a 60-min rebaseline session during which mice were reinforced for nosepoking on an FR1 schedule. During a 30-min test session, mice were presented with 3-min PE and NPE stimuli with an 8-min ISI. Mouse behavior was video recorded and the duration of freezing behavior was estimated using FreezeScan software (CleverSys Inc., Reston, VA, USA; Buhusi et al., 2017).

### c-Fos Immunostaining

To evaluate neuronal activation, we performed c-Fos immunostaining using standard procedures (Buhusi et al., 2016). Two hours after the start of the test session 5–9 mice in each group were deeply anesthetized and transcardially perfused with a paraformaldehyde solution (4% in 0.1 M phosphate buffer, pH 7.4). Brains were collected and sectioned on a vibrating microtome (VT1200S, Leica, Germany). Free-floating brain sections (50 μm) were incubated with a blocking and permeabilization solution (5% donkey serum, 0.3% Triton X-100 in PBS) for 2 h and then incubated overnight at 4°C with the c-Fos primary antibody (Cell Signaling Technologies, 1:300 dilution). The sections were rinsed in PBS, 0.1% Tween-20 and incubated for 2 h with Alexa488-conjugated donkey anti rabbit secondary antibody and NeuroTrace 530/615 (Fisher Scientific/Invitrogen, Carlsbad, CA, USA). NeuroTrace neuronal labeling was used to identify the neuroanatomical regions of interest. The sections were rinsed in PBS before mounting with Prolong Gold (Fisher Scientific/Invitrogen, Carlsbad, CA, USA).

### Neuronal Activation Analysis

Fluorescence images were acquired on a Zeiss LSM710 laser scanning confocal microscope using appropriate filter sets. Analysis of neuronal activation was performed by counting c-Fos-positive nuclei, in same-size areas in two sections/region of interest/mouse in the following areas of interest: *prelimbic cortex* (PrL: Bregma 2.1–1.54), *ventral hippocampus* (vHipp: Bregma −2.92 to −3.40), *nucleus accumbens shell* (Acb-shell: Bregma 1.78–1.1), and *nucleus accumbens core* (Acb-core: Bregma 1.78–1.10; Franklin and Paxinos, 2008), by two independent observers unaware of genotype. Neuronal activation in each region was averaged over observers and subjected to statistical analyses.

### Statistical Analyses

The estimated duration of freezing behavior in the first 60 s of the presentation of the PE and NPE stimuli during the conditioning and test sessions was subjected to mixed ANOVAs with between-subjects variables stress (S, NS) and genotype (HET, WT), and within-subjects variable pre-exposure (PE, NPE), followed by *post hoc* analyses. The latency to freeze (to the context) during the conditioning and test sessions was subjected to mixed ANOVAs with between-subjects variables stress (S, NS) and genotype (HET, WT), and within-subjects variable session (conditioning, test), followed by *post hoc* analyses. The difference in freezing between NPE and PE, the number of rewards and nosepokes during the test session, and the neuronal activation (c-Fos+ cell counts in each brain region) were subjected to two-way ANOVAs with factors stress (S, NS) and genotype (HET, WT), followed by LSD *post hoc* analyses. To further explore data, results were collapsed over stress and/or genotype (to yield larger groups), and correlational analyses were conducted between LI (the difference in freezing to the NPE and PE stimuli) and neuronal activation (c-Fos+ cell counts) for Acb-shell and Acb-core. All statistical analyses were conducted at an alpha level 0.05.

## Results

### Latent Inhibition

**Figure 1:**
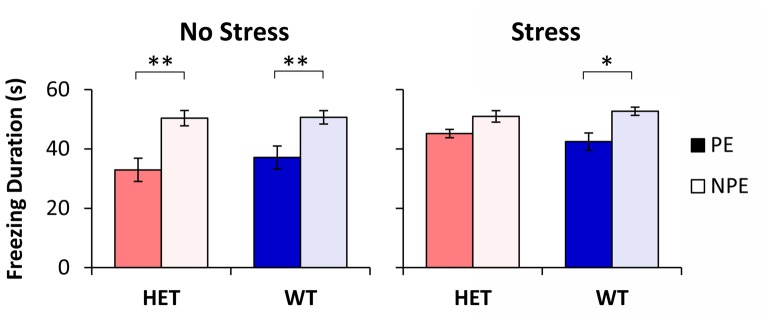
Latent inhibition (LI) by stress and genotype. Average duration of freezing (±SEM) to the pre-exposed (PE) and non-pre-exposed (NPE) stimulus in glial-derived neurotrophic factor (GDNF) heterozygotes (HET) and wild type (WT) littermate controls under no-stress (left) and chronic unpredictable stress (CUS; right). A significant LI (significantly larger freezing to NPE than PE) was observed in all groups except in stressed GDNF HET mice. *p < 0.05; **p < 0.01.

The average freezing duration during the PE and NPE stimuli during the test session is shown in Figure 1. Analyses indicated a main effect of pre-exposure (*F*_(1,48)_ = 52.96, *p* < 0.01), suggesting that mice froze longer during the NPE stimulus than during the PE stimulus (LI). However, LI was not expressed equally in all groups: Analyses indicated a significant main effect of stress (*F*_(1,48)_ = 5.51, *p* < 0.05), suggesting that NS mice showed more LI than S mice. Furthermore, analyses indicated a significant pre-exposure × stress interaction (*F*_(1,48)_ = 5.34, *p* < 0.05), suggesting that stress increased freezing to the PE stimulus, but not to the NPE stimulus. A *post hoc* LSD test indicated a significant difference in freezing between NPE and PE in all NS mice (all *p*s < 0.01), and in the stressed WT mice (*p* < 0.05), but not in stressed GDNF HET mice (*p* > 0.05), indicating that all mice showed LI except stressed GDNF HET mice.

Analyses of LI, the difference in freezing between NPE to PE, supported the above results: a factorial ANOVA with factors stress and genotype indicated a significant main effect of stress (*F*_(1,48)_ = 5.34, *p* < 0.05). LI was large in NS mice (13.5 ± 3.9 s in WTs, and 17.4 ± 3.5 s in HETs), but it decreased in stressed mice (10.3 ± 2.9 s in WTs, and 4.8 ± 2.5 s in HETs). A *post hoc* LSD test indicated that LI did not change with stress in WT mice (*p* > 0.05), but was significantly decreased in stressed GDNF HET mice relative to no-stress mice (*p* < 0.05). Taken together, these results provide support for a “two-hit” model under which environmental factors (stress) potentiate the effect of genotype to reveal the disruption of LI in stressed GDNF HET mice but not in the other groups.

### Unconditioned Freezing

To evaluate the hypothesis that the difference in freezing to PE and NPE stimuli in Figure 1 may be due to the intrinsic (unconditioned) differences in freezing to the two stimuli, we performed analyses of freezing behavior to the PE and NPE stimuli in the conditioning session, before these stimuli were paired with footshock. These analyses failed to indicate any main effects of stimulus (PE/NPE; *F*_(1,48)_ = 2.07, *p* > 0.05), genotype (*F*_(1,48)_ = 1.49, *p* > 0.05), stress (*F*_(1,48)_ = 0.15, *p* > 0.05), or any interactions (all *F*s < 1.99, *p* > 0.05), suggesting no differences in unconditioned freezing to the PE and NPE stimuli, irrespective of genotype and stress condition. This result indicates that the differences in freezing between groups in Figure 1 are not due to differences in unconditioned freezing, but reflect differences in conditioned freezing (associability/learning), thus describing true differences in LI.

### Reactivity to Shock

Another possibility is that stressed GDNF HET mice became more reactive to shock than the other groups. To evaluate this hypothesis we followed three lines of evidence: first, a *post hoc* LSD test of the duration of freezing during the test session (see “Latent Inhibition” Section) failed to indicate differences between genotypes in duration of freezing to the NPE stimulus (all *p*s > 0.05; see Figure 1); same analyses also failed to indicate differences in duration of freezing to the NPE stimulus between unstressed and stressed mice for each genotype (all *p*s > 0.05; see Figure 1). Taken together, these analyses suggest that all mice learned similarly about the NPE stimuli, thus making it unlikely that they had different reactivity to shock.

Second, analyses of the latency to freeze in the conditioning session (before exposure to shock) and in the test session (after exposure to shock) failed to indicate any effects of session (*F*_(1,48)_ = 0.74, *p* > 0.05), genotype (*F*_(1,48)_ = 2.21, *p* > 0.05), stress (*F*_(1,48)_ = 2.31, *p* > 0.05), or any interactions (all *F*s_(1,48)_ < 3.27, all *p*s > 0.05), suggesting that the propensity to freeze in the given context did not change after exposure to shock, and did not vary with stress and genotype, thus making it unlikely that mice differed in their reactivity to shock.

Finally, analyses of the number of rewards earned, and number of nosepoke responses emitted, during the test session failed to indicate any effects of genotype (all *F*s_(1,48)_ < 0.02, all *p*s > 0.05), stress (all *F*s_(1,48)_ < 3.61, all *p*s > 0.05), or stress × genotype interaction (all *F*s_(1,48)_ < 0.18, all *p*s > 0.05), suggesting that mice responded similarly, and earned food similarly, irrespective of stress and genotype. These results indicate that stressed GDNF HET mice nosepoked and were rewarded similarly with the other mice, thus making it unlikely that the absence of LI in stressed GDNF HET mice is due to these mice being more reactive to shock than the other mice.

In summary, the three lines of evidence indicate that all groups were similar in nosepoking, earning food, learning about the context and learning about the NPE stimulus, suggesting that all groups were similarly reacting to and/or learning about the footshock. Nevertheless, groups differed in their freezing to the PE stimulus (see Figure 1): Freezing to the PE stimulus was significantly smaller than freezing to the NPE stimulus in all unstressed mice and in the WT stressed mice, but increased (to levels not significantly different than freezing to the NPE stimulus) only in the stressed GDNF HET mice, indicative of impaired LI.

### Neuronal Activation

**Figure 2:**
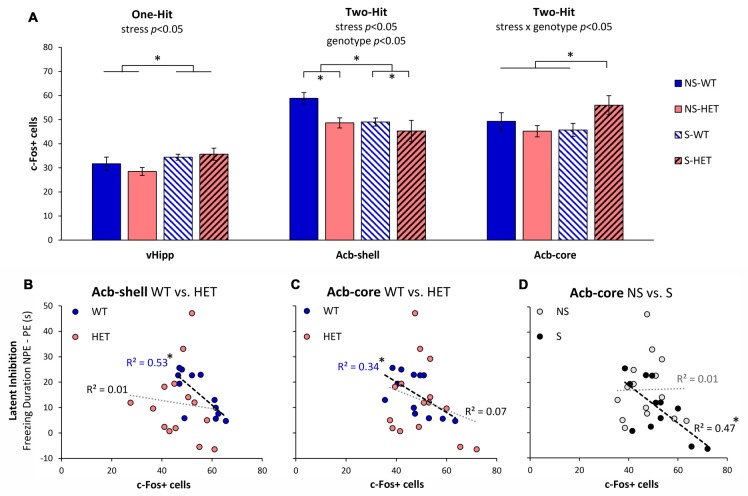
Neuronal activation during LI testing. **(A)** Average c-Fos+ cell counts (±SEM) in ventral hippocampus (vHipp), nucleus accumbens shell (Acb-shell), and nucleus accumbens core (Acb-core) in the stress (S) and no-stress (NS) GDNF-deficient mice (HET) and WT littermate controls. Analyses indicated different patterns of effects of stress and genotype on neuronal activation in these brain regions: vHipp activation was affected only by stress (one-hit), Acb-shell activation was independently affected by stress and genotype (independent two-hit), while Acb-core activation was affected by the interaction stress × genotype (two-hit interaction). **(B–D)** Correlations between LI (difference in freezing duration to the NPE, and PE, stimuli) and neuronal activation (number of c-Fos+ cells) in Acb-shell **(B)** and Acb-core **(C,D)** when data are collapsed across stress **(B,C)** or genotype **(D)**. *p < 0.05.

As previously shown in Sotty et al. (1996), we have assessed neuronal activation during LI through analyses of expression of the immediate early gene c-Fos in brain regions known to be relevant to LI through lesion or pharmacological studies (Yee et al., 1995; Pouzet et al., 2004; Schiller and Weiner, 2004; Gal et al., 2005; Schiller et al., 2006; Ouhaz et al., 2014). Figure 2A indicates three different patterns of neuronal activation: first, neuronal activation in vHipp was affected only by stress (*F*_(1,22)_ = 5.39, *p* < 0.05), but not by genotype (*F*_(1,22)_ = 0.21, *p* > 0.05), or interactions (*F*_(1,22)_ = 1.11, *p* > 0.05). Second, Acb-shell was independently affected by stress (*F*_(1,22)_ = 4.55, *p* < 0.05) and genotype (*F*_(1,22)_ = 5.06, *p* < 0.05), but not by the stress × genotype interaction (*F*_(1,22)_ = 1.08, *p* > 0.05). Third, Acb-core activation was not affected by neither stress alone (*F*_(1,23)_ = 1.14, *p* > 0.05) nor genotype alone (*F*_(1,23)_ = 0.83, *p* > 0.05), but was significantly affected by a stress × genotype interaction (*F*_(1,23)_ = 4.80, *p* < 0.05). A *post hoc* LSD test indicated that Acb-core activation was significantly increased in GDNF-HET mice relative to the other groups (*p* < 0.05). Finally, in the present study, PrL neuronal activation was not affected by either stress, genotype, or their interaction (all *F*s_(1,27)_ < 0.31, all *p*s > 0.05). These results indicate that various brain regions relevant to LI are differentially affected by stress, genotype, and their interaction, thus supporting a complex “two-hit” stress × genotype model.

To further understand the effect of stress and genotype on these brain regions, we evaluated the relationship between LI (the difference in freezing duration to NPE and PE stimuli) and neuronal activation (number of c-Fos+ cells) in the nuclei that control the behavioral output (Schmajuk et al., 1997; Weiner, 2003), Acb-shell and Acb-core, as shown in Figures 2B–D. Figures 2B,C shows the relationship between LI and neuronal activation in Acb-shell (Figure 2B) and Acb-core (Figure 2C) in WT and HET mice when data are collapsed over the stress variable. Consistent with previous studies (Sotty et al., 1996), Figure 2B indicates that irrespective of the stress condition, LI correlated with Acb-shell activation in WT mice ($R_{\left(10\right)}^{2}$ = 0.53, *p* < 0.05), but not in GDNF HET mice ($R_{\left(12\right)}^{2}$ = 0.01, *p* > 0.05). Similarly, Figure 2C indicates that irrespective of the stress condition, LI correlated with Acb-core activation in WT controls ($R_{\left(10\right)}^{2}$ = 0.34, *p* < 0.05), but not in GDNF HET mice ($R_{\left(13\right)}^{2}$ = 0.07, *p* > 0.05). Indeed, as shown in Figure 2A, stress determined a decrease in Acb-shell activation and an increase in Acb-core activation in stressed GDNF HET mice, such that stressed GDNF HET mice, but not stressed WT mice, showed impaired LI (Figure 1).

Moreover, Figure 2D shows that the relationship between LI and Acb-core activation differs in no-stress (NS) and stress (S) mice when data are collapsed over genotype: Irrespective of genotype, LI correlated with Acb-core activation in S mice ($R_{\left(10\right)}^{2}$ = 0.47, *p* < 0.05), but not in NS mice ($R_{\left(13\right)}^{2}$ = 0.01, *p* > 0.05). Indeed, Figure 2A indicates that there was no significant difference in Acb-core activation in S and NS WT, which showed LI (Figure 1), while stressed GDNF HET mice showed an increase in Acb-core activation (Figure 2A), and failed to show LI (Figure 1). In summary, all three patterns in Figures 2B–D contributed to the disruption of LI in stressed GDNF HET mice, and to the significant LI in the other mice, as shown in Figure 1.

## Discussion

Using an “on baseline” within-subject CER LI procedure developed in our lab (Buhusi et al., 2017), the current study found that WT mice showed LI, consistent with previous findings (Gould and Wehner, 1999). Additionally, results indicated that GDNF HET mice in C57BL/6J background showed LI under baseline, no-stress conditions. However, after exposure to a CUS regimen, GDNF HET mice failed to show LI, while WT littermates continued to show LI. These results are unlikely to be due to differences in unconditioned freezing to the two stimuli, or to differences in reactivity to shock, as all mice froze similarly to the two stimuli (before they were paired with shock), learned similarly about the NPE stimulus and context, nosepoked similarly and were rewarded similarly in the FR1 task. Further studies are required to evaluate whether altered LI as a consequence of the stress × GDNF-deficit interaction reflects anomalies in either acquisition or expression of LI.

Neuronal activation analyses (c-Fos+ cell counts) in brain regions involved in LI indicated that in some brain regions activity was affected solely by stress (vHipp), while in others it was affected by both stress and genotype (Acb-shell) or their interaction (Acb-core). Our results revealing the combined effects (including an interaction) of stress and genetic factors on neuronal activation in the Acb support current neurobiological (Weiner, 2003) and neuro-computational models (Schmajuk et al., 1997, 1998; Buhusi et al., 1998) of LI.

### Neural Substrates of Latent Inhibition

**Figure 3:**
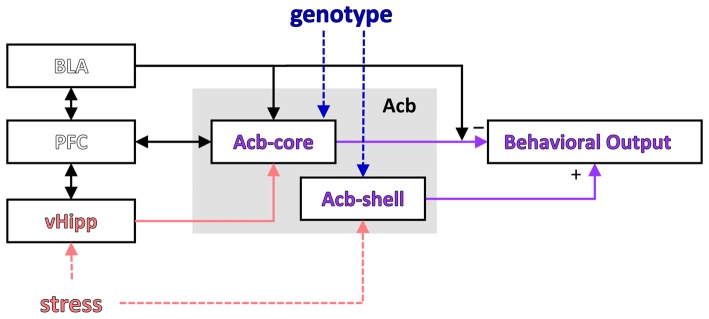
Modulation of a putative LI circuit by stress or the GDNF genotype. A putative circuit for LI (modified after Schmajuk et al., 1997; Weiner, 2003) indicating the brain regions where activity was affected by stress and/or genotype. PFC, prefrontal cortex; vHipp, ventral hippocampus; Acb, nucleus accumbens; Acb-core, nucleus accumbens core; Acb-shell, nucleus accumbens shell.

LI is a phenomenon observed both in humans and other species by which repeated presentation of a neutral stimulus with no consequences reduces its future associability relative to learning about other novel stimuli (Lubow and Moore, 1959; reviewed in Lubow, 1989). Most theories relate LI to selective attention, i.e., during pre-exposure of an inconsequential CS, the animal or participant learns not to attend to it (Pearce and Hall, 1980; Lubow and Gewirtz, 1995; Schmajuk et al., 1997). Weiner and Feldon (1997) suggested a “switching” theory in which the nucleus accumbens plays a major role (see Figure 3): during pre-exposure a CS-noUS association is learned (with the involvement of the hippocampus), after which the new CS-US association requires switching controlled by the core of the nucleus accumbens (with the shell having a modulatory role; Weiner, 2003; Gray and Snowden, 2005). According to this theory, LI is disrupted by the change of context between pre-exposure and conditioning (Lubow et al., 1976), suggesting that the hippocampus may be important for detecting the mismatch.

Interestingly, the results of our study support a computational model suggesting that LI is affected by the interaction between environmental stimuli and brain insults (Schmajuk et al., 1997; Buhusi et al., 1998; see Figure 3). In this neural network model LI depends on the novelty of the PE and NPE stimuli relative to the context (computed in the VTA and modulating activity in the accumbens), which relies on learned associations between stimuli (which in turn depend on normal hippocampal function). Thus, according to this model, current data could be explained by genetically-induced alterations in brain function combined with environmental factors (e.g., decreased expression of GDNF and inability to up-regulate GDNF expression in the hippocampus during stress), which interact to alter novelty computation and activity in the accumbens, and impair LI in stressed GDNF HET mice.

Although Weiner’s “switching model” and Schmajuk’s “novelty” model describe activity in the same network (investigated in this article, and shown in Figure 3), the interpretation of neuronal activity and its behavioral correlates (Figure 2 in this article) is very different in the two models: Weiner’s model interprets neuronal activity as reflecting changes in switching, while Schmajuk’s model interprets neuronal activity as reflecting changes in novelty. Further studies are required to evaluate and differentiate these models, although it is notable that Schmajuk’s “novelty” model already incorporates and addresses environment × novelty interactions (Buhusi et al., 1998), thus possibly addressing the data from the current study.

As suggested by the above theories, multiple studies have shown that the Acb and the hippocampus are indeed key structures in LI acquisition and expression. Lesion studies revealed opposing roles of Acb-shell and core in LI: lesions of the Acb-shell impair LI (Weiner et al., 1999), while lesions of Acb-core or Acb-shell+core are associated with persistent LI (Weiner et al., 1999; Gal et al., 2005). Our results showing that stressed GDNF HET mice which have impaired LI also have decreased c-Fos+ cell counts in the Acb-shell and increased neuronal activation in the Acb-core are consistent with these previous findings.

Hippocampal lesions revealed maintenance of LI, but loss of context specificity of the CR and LI (Good and Honey, 1991; Honey and Good, 1993; Coutureau et al., 1999), however LI is disrupted after ventral hippocampal (vHipp)/ventral subiculum (vSub) NMDA receptor activation (Pouzet et al., 2004; Lodge and Grace, 2008). Our findings that stress increases c-Fos+ cell counts in the ventral hippocampus in the LI procedure also support a role for the increased vHipp activity in the disruption of LI.

The involvement of the prefrontal cortex, which is bi-directionally connected with the hippocampus and amygdala and projects to the Acb (Del Arco and Mora, 2008, 2009; see Figure 3), has also been evaluated in relation to LI, with mixed results: excitotoxic lesions of the medial prefrontal cortex did not affect LI (Lacroix et al., 1998), while catecholaminergic depletion within the medial prefrontal cortex enhanced LI (Nelson et al., 2010). In our study, assessment of neuronal activation in the prelimbic cortex (part of the medial prefrontal cortex) during the LI task has revealed no differences between experimental groups, consistent with the Lacroix et al.’s (1998) study. The absence of differences in the prelimbic cortex activation between experimental groups in the LI task further suggests that in our study the changes in neuronal activity were not general, but were rather specific to certain brain areas.

### Stress, Latent Inhibition and Schizophrenia

Chronic stress induces changes in gene expression (including an up-regulation of GDNF expression in resilient individuals, see Uchida et al., 2011), and alters neuronal morphology and function in many brain regions, including regions relevant for the acquisition and expression of LI. For example, after stress pyramidal neurons in the cortex and hippocampus exhibit altered dendritic and spine morphology and decreases in spine density (Cook and Wellman, 2004; Maras and Baram, 2012; Leuner and Shors, 2013; McEwen and Morrison, 2013). Interestingly, CUS is associated with increased dendritic complexity in the Acb-core, while decreased dendritic complexity is found in the Acb-shell (Taylor et al., 2014); these results may explain the differences in neuronal activation observed in the two regions of Acb in our study.

Stress also induces alterations in DA neurotransmission, which is particularly important for the acquisition and expression of LI (Young et al., 2005; Weiner and Arad, 2009): Rats exposed to CUS have a decreased DA output in the Acb-shell accompanied by a decrease in the number of DAT binding sites (Scheggi et al., 2002). Indeed, stress was shown to attenuate LI in humans (Braunstein-Bercovitz et al., 2001) or rats (Hellman et al., 1983), although, in some cases, it may potentiate it (Melo et al., 2003). Knapman et al. (2010) reported a reduction in LI in mice highly reactive to stress supporting our own observation that genetic factors are major contributors to vulnerability to stress: in our study only stressed GDNF HET mice, but not stressed WT littermates, failed to show LI.

In vulnerable individuals chronic stress can precipitate psychiatric disorders (Bale, 2006; Deppermann et al., 2014; Nestler et al., 2016), including schizophrenia (Aiello et al., 2012; Holtzman et al., 2012, 2013). SZ is a chronic neuropsychiatric disorder, characterized by delusions, hallucinations, disorganized behavior and speech, and attentional control deficits, symptoms that can lead to severe impairments in adaptive function and social integration (van Os and Kapur, 2009). Interestingly, disrupted LI is an important feature in SZ, particularly in drug-naïve patients or during acute episodes (Baruch et al., 1988; Gray et al., 1995; Williams et al., 1998; Rascle et al., 2001). Abnormal LI in SZ may be explained by patients having selective attention deficits and continuing to attend irrelevant stimuli (Lubow, 1989), having a hyperactive “switching” mechanism (Hemsley, 1993), or a hyperactive novelty system (Schmajuk et al., 1997; Buhusi et al., 1998). Impaired LI is thought to underlie the positive symptoms of SZ (Morris et al., 2013). Drug treatment, using either typical or atypical neuroleptics, leads to normalization of LI (Leumann et al., 2002), possibly by modifying neuronal activation threshold in specific brain areas.

### GDNF and Schizophrenia

Several neuropsychiatric diseases are associated with alterations in GDNF expression levels (Rosa et al., 2006; Tseng et al., 2013; Tunca et al., 2015); serum levels of GDNF and other neurotrophic factors are also modified by treatment (Zhang et al., 2008). Interestingly, although serum GDNF levels are decreased in SZ (Tunca et al., 2015), genetic association studies between GDNF and SZ produced mixed results (Lee et al., 2001; Michelato et al., 2004; Williams et al., 2007). However, GDNF-receptor genes GFRA1, GFRA2 and GFRA3 are located in chromosomal regions with suggestive linkage to SZ. A recent study (Souza et al., 2010) found evidence for genetic associations between GFRA1 and 3 and schizophrenia, as well as evidence for GFRA2 variants modulating the therapeutic response to clozapine. Our results support a role for the GDNF signaling pathway and its interaction with stress in the development of abnormal behaviors relevant to SZ and other mental disorders.

## Conclusion

This study identifies a disruption of LI in stressed GDNF-deficient mice, providing strong evidence for a role of chronic stress in LI alterations in individuals with particular genetic vulnerabilities. The disruption of LI may be the result of small changes in neuronal function or connectivity related to genotype which is potentiated as a result of chronic stress. Our results add LI to the list of behaviors affected by chronic stress and support a role for GDNF deficits in stress-induced pathological behaviors relevant to schizophrenia and other psychiatric disorders.

## Author Contributions

MB: experimental design and immunostaining and imaging. CKB and CVB: behavior. MB and CVB: data analysis and wrote the article.

## Conflict of Interest Statement

The authors declare that the research was conducted in the absence of any commercial or financial relationships that could be construed as a potential conflict of interest.
